# Veterinary Anatomy

**Published:** 1910-11

**Authors:** 


**Veterinary Anatomy.** By **Septimus Sisson**, S.B., V.S., Professor of Comparative Anatomy, Ohio State University, College of Veterinary Medicine. Octavo of 826 pages, 528 illustrations. Philadelphia and London: W. B. Saunders Company, 1910. (Cloth, $7.00; half morocco, $8.50, net prices.)

The lack of a modern and well illustrated book on the structure of the principal domestic animals has been acutely felt for a long time by teachers, students, and practitioners, of veterinary medicine. The work here offered is the expression of a desire to close this gap in our literature. The experience of the author during the last ten years has demonstrated that many of the current descriptions of the organs in animals contain the same sort of errors as those which prevailed in regard to similar structures in man previous to the adoption of modern methods of preparation. In this work an attempt is made to eliminate some terms which do not appear to fulfil any useful purpose and others which are clearly erroneous or otherwise undesirable, and to offer a textbook of convenient size for the student and a guide of ready reference for the practitioner. J. A. R.

